# Model based estimation of population total in presence of non-ignorable non-response

**DOI:** 10.1371/journal.pone.0222701

**Published:** 2019-10-10

**Authors:** Shakeel Ahmed, Javid Shabbir

**Affiliations:** Department of Statistics Quaid-i-Azam University, Islamabad, Pakistan; Universidade Federal Fluminense, BRAZIL

## Abstract

The problem of handling non-ignorable non-response has been typically addressed under the design-based approach using the well-known sub-sampling technique introduced by Hansen and Hurwitz [1946, Journal of the American Statistical Association, Vol 41(236), Page 517- 529]. Alternatively, the model-based paradigm emphasizes on utilizing the underlying model relationship between the outcome variable and one or more covariate(s) whose population values are known prior to the survey. This article utilizes the model relationship between the study variable and covariate(s) for handling non-ignorable non-response and obtaining an unbiased estimator for the population total under the sub-sampling technique. The main idea is to combine the estimates obtained from the sample on first call and the sub-sample from second call using separate model relationships. The contribution of this paper helps us in providing unbiased estimates with an improved efficiency under model-based paradigm in presence of non-ignorable non-response. The provided method is more economical than the available estimators under callback methods as we are working sub-sampling and also increase response rate as a stronger mode of interview is employed for data collection. A numerical study using Monte Carlo is presented to illustrate the behavior of the proposed and the efficiency comparison.

## 1 Introduction

In statistical investigations, once data collection is completed, one has to bear some, perhaps a considerable amount of non–response. Although a significant resource can be employed to improve data collection process to avoid the non-response about 20% non–response rate is commonly accepted. Item non-response occurs when one or more questions in the questionnaire are left unanswered during the survey. While a unit non-response occurs when one or more unit(s) do not response at all or are missing. Non–response in sample surveys leads to non-sampling error in estimation of the population parameters and yields biased estimates which ultimately spoils inference about the population of interest. When non-response occurs completely at random then the best way to deal with is to impute the projected values of the outcome variable corresponding to non-respondents. On contrary, when non-response factor (e.g, age, sex or/and income status etc.) is correlated with the outcome variable then the usual imputation methods fail to cope with the situation. In such situations, the population parameters and the behavior of the population may differ among the responding population (respondents) and the responding populations (non-respondent).

There are several approaches for checking whether there is a difference between the populations of respondents and non-respondents and evaluating potential bias due to non–response: (i) specific follow-up of non-respondents and (ii) analysis of the characteristics of respondents and non-respondents which are known prior to survey. [1] used demographic information (education, age, employment status, state of residence, field of employment etc.) to compare the respondents and the non-respondents. Information regarding non-respondents may come from previous surveys of same population (in the case of longitudinal surveys or with rotation groups) or by using some external data sources (e.g. administrative data etc.). [2] suggested a method for adjustment of non-ignorable non-response in studies involving one or more additional attempts to contact initial non-responders. [3] worked on changing in survey estimates as a function of additional calls under the similar protocol as well as under a different protocol. [4] considered the use of level-of-effort paradata to model the mechanism of non–response in surveys and for adjusting non–response bias, specially bias that is not missing at random (NMAR) or non-ignorable. The approach was based on unconditional maximum likelihood estimation model that adapted and extended the prior work to cope with the complexities encountered in large-scale surveys.

For similar situation, [5] examined whether non-participation in a census-based health study was related with poorer health status, using the Hordaland Health Study conducted in western Norway in 1997-1999. They aimed to determine whether health problems were over–represented in nonparticipants and to explore the consequences of participation bias on relation between outcomes and exposures. Statistical techniques for dealing with non–ignorable non–response based on a propensity–to–respond score has been developed by [6] assuming both item as well as unit non–response. Moreover, [7] proposed an approach of increasing blood supply by collecting blood more frequently from the selected donors for studying the relationship between ageing the population and blood transfusion. The primary aim of their proposed INTERVAL trial was to observe whether donation intervals can be acceptably and safely decreased to optimize blood supply while maintaining the health status of donors. The health status of a cohort of 1991 Gulf War veterans was periodically assessed by [8]. They compared various health outcomes of veterans with those of their peers in military who were not posted to the Gulf. Another example in which one can make utilization of sub-sampling method can be found in [9], where missing data and incomplete randomized interventions were common. These problems complicate the analysis as well as interpretation of controlled randomized trials (CRT), and are rarely handled well in practice. [10] modeled the non-response probabilities as logistic functions of the survey variable and related covariates in the survey with callback. They proposed maximum likelihood semi-parametric estimators of the parameters in the response probabilities. They further proposed, an efficient estimator for the mean of the study variable using the estimated response probabilities. The method was employed to data taken from the Singapore Life Panel Survey, a survey of health spending utilizing a census-based sample of individuals 50-70 years old, assuming that non-response was related to the health status.

In real surveys, as discussed in above cited works, non-response occurrence is not missing at random (NMAR) or, in other word, it is non-ignorable. When the occurrence of non-response in sample survey is related to the outcome of the survey, a valid statistical inference about the target population is quiet difficult. One can make efficient utilization of the sub-sampling method instead of call back. To fill this gap, [11] introduced a well known procedure for sub-sampling (follow-up) the non-respondents. The method includes sub-sampling a portion of non-respondents from the first sample with the assumption that some stronger mode of interview is applied for the purpose of sub-sampling non-respondents, consequently, all persons give full response on second call. On the basis of sub-sampling procedure introduced by [11], many authors including [12, 13], [14] and [15] worked on mean estimation under designed-based approach ignoring model relationship between the study variable and the known covariates. [16] suggested Hansen and Hurwitz [11] type estimator under Bayesian paradigm using squared error loss function (SELF). Later on [17] considered Bayesian approach of estimation under a general model using [11] technique. In survey sampling, usually one assumes the population as a finite collection of distinct and countable units. The measurements on the variable under investigation in the population are considered to be non-stochastic. The focus lies in estimation of population parameters i.e. functions of the population measurements on the study variable in the population (such as mean, total, proportion etc), which are also non-stochastic consequently. A sample is considered just as a smaller collection of population units and inference is carried out typically under the probability distribution formed by the random mechanism employed to draw the sample, which is termed as sampling design (S.D). Desirable properties of the estimators such as unbiasedness and efficiency are established by averaging out the values of the estimators over all possible samples.

While in model-based inference, a population is considered as a collection of realizations of a set of stochastic variables with a specified but unknown mean and a specified variance (usually assumed to be known). While a sample is a collection of identically distributed and independent variables for some fixed S.D. The parameters to be estimated are characteristics of the distribution of the original stochastic variables such as mean, and lower order moments, which are assumed to be constant quantities under the frequentist point of view.

Under model-based statistical inference [18] worked on estimation of a finite population mean. [19] attempted to obtain optimal model-unbiased estimators of the population mean and total using least square (LS) estimation method and the well known Gauss-Markov Theorem (GMT) assuming linear population model. [20] introduced the linear least-square prediction approach for estimation of finite population parameters under two-stage sampling. Other related works on estimation of mean and total under model-based approach can be found in [21], [22], [23], [24], [25], [26], [27] and [28]. [29] adapted mixed model prediction in small areas. Furthermore, [30] compared the model-based approach with model-assisted approach. For an updated comparison of the model-based and the designed based frameworks see [31]. A detailed review of the model-based estimation can also be found in [32]. As we already mentioned that the presence of non-response in sample surveys not only creates problem of small sample size but also spoils the inference when the behavior (underlying model relationship) of the population of respondents and non-respondents are different.

In current article, a model unbiased linear predictor for the population total in presence of non-ignorable non-response is proposed assuming unit non-response. The sub-sampling technique introduced by [11] is used to obtain samples under a fixed sampling design. We provide a revision of model-based approach for estimation of superpopulation total in Section 2. Our proposed estimator and its properties under assumed model are given in Section 3. Some shortcomings of the proposed estimation technique and their possible solutions are discussed in Section 4. A numerical study with real data set and a Monte Carlo simulation are respectively provided in Sections 5 and 6. A discussion with concluding remarks is given in Section 7.

## 2 Model-based estimation of population total

Consider a finite population of *N* distinct units *U* = {1, 2, ‥*i*‥, *N*}. Let ***y*** = (*y*_*i*_, *i* ∈ *U*) be the vector of the realized values of a stochastic vector ***Y*** = (*Y*_*i*_, *i* ∈ *U*) of order *N* × 1 and ***x*** = (*x*_*ij*_, *i* ∈ *U*, *j* = 0, 1, 2, …, *p*) be a matrix of (p+1) auxiliary variables whose values are assumed to be known for every unit in *U*. We start with multiple linear regression model ***Y*** = ***xβ*** + ***ϵ***, where ***β*** = (*β*_0_, *β*_1_, …., *β*_*p*_)^*T*^ and ***ϵ*** = (*ϵ*_1_, *ϵ*_2_, …., *ϵ*_*N*_)^*T*^ be the vectors of regression coefficients and the random error terms respectively. Let *s* = {1, 2, 3, …, *n*} be a member of *S* of all possible samples of size *n* that can be drawn from *U* using some S.D. Further, the random vector of the study variable ***Y***, the known auxiliary matrix ***x*** and the random error vector ***ϵ*** are splited into sampled (*s*) and non-sampled $\left(\bar{s}\right)$ as: $Y={\left(Y_{s};Y_{\bar{s}}\right)}^{T}$, $x={\left(x_{s};x_{\bar{s}}\right)}^{T}$ and $\epsilon ={\left(\epsilon_{s};\epsilon_{\bar{s}}\right)}^{T}$, where $\bar{s}=U-s$. The population total *T*_*y*_ (which is assumed to be random under model-based approach) is expressed as $T_{y}=W_{s}^{T}Y_{s}+W_{\bar{s}}^{T}Y_{\bar{s}}$, where ***W*** = (*w*_*i*_, *i* ∈ *U*) is the vector containing 1’s for every units in population. For obtaining population mean ***W*** are taken as vector of 1/*N* for all units. Optimal values of $w_{i}^{\prime }s$ are found by minimizing the prediction variance which is considered as good practice in model based approach [32]. For further statistical inference about the estimated parameter assumption of normally distributed error term is also necessary specially in case of small sample sizes. After observing ***y***_*s*_ as the realized values of ***Y***_*s*_ the problem is to predict sub-vector $Y_{\bar{s}}$ using the information contained in the sample and the auxiliary information through model relationship between the study variable and the auxiliary variable(s). Under linear population model, a predictor for $Y_{\bar{s}}$ is $x_{\bar{s}}b$, where the vector ***b*** = (*b*_0_, *b*_2_, …., *b*_*p*_)^*T*^ is the solution of the normal equations $x_{s}^{T}x_{s}b=x_{s}^{T}y_{s}$ which is obtained by minimizing the sum of squared residuals. The model-based estimator given in [33] is

$$\begin{array}{c} {\hat{T}}_{y}=W_{s}^{T}y_{s}+W_{\bar{s}}^{T}x_{\bar{s}}b. \end{array}$$ (1)

Note that the total estimator given in (1) works only when error terms are iid with zero mean and constant variance [27]. ${\hat{T}}_{y}$ posses all the properties with respect to the model as the predictor of $y_{\bar{s}}$ does [18, 19]. When all OLS assumptions fulfill the estimator ${\hat{T}}_{y}$ is model unbiased with the model-variance after averaging over all possible sample of same S.D.

$$\begin{array}{c} E_{D}\{V_{m}\left({\hat{T}}_{y}\right)\}=\sigma^{2}E_{D}[W_{\bar{s}}\{x_{\bar{s}}{\left(x_{s}^{T}x_{s}\right)}^{-1}x_{\bar{s}}^{T}+I_{N-n}\}W_{\bar{s}}^{T}], \end{array}$$ (2)

where the subscript *D* is used to show that the expectation is applied with respect to S.D and ***I***_*N*−*n*_ is the identity matrix of order (*N* − *n*) × (*N* − *n*). Setting *p* = 0 the linear regression model reduces to homogeneous population model i.e. ***Y*** = ***x***_0_***β***_0_ + ***ϵ***, where ***x***_0_ is vectors of 1’s. Care should be taken while selecting a suitable set of predictors which comes under the domain of variable selection (inclusion and exclusion) [34]. Moreover, when variance of the error term depends on some function of the auxiliary variable(s), weighted least square (WLS) estimator is preferred for estimating ***β*** as alternative to OLS. Moreover, if the number of regressors exceeds number of observations in the sample then ridge regression is preferred [27, 35]. We discuss these problems for our proposal later in Section 4.

## 3 Model-based estimation of population total in presence of non-response

In voluntary surveys, a common threat to the validity of the survey estimates is the problem of non–response. Different surveys possess different response rates, the surveys that ask questions which seem interesting and relevant to the respondents are tend to achieve the highest response rates. In recent years, response rates have been declined even in popular surveys, and, as a consequence, worries about non-response bias have been increased. As we discussed in introduction section that non-response is considered as problematic only if the population of non-respondents is an informative sample of the total sample. Unfortunately, this appears almost in majority of practical applications. In household surveys, for instance, there is a lot of evidence that non-respondents are often younger than respondents, and that women are more likely to persuade to take part than men. Similarly, response rates are also tend to be lower in deprived areas than the areas with abundance of facilities. All of these examples show that the pattern of achieved samples for surveys mostly do not reflect the population that is meant to represent very well. These surveys typically may over-represent women, and the persons elder than certain age. And often under-represent those living in less developed cities and deprived areas. When values of such demographic variable(s) are known for whole target population, we can stratify the population as the respondents and the non-respondents. The problem is then to choose a variable which more accurately stratifies the population as respondents and non-respondents. Suppose that ***R*** is a stratification vector defined as ***R*** = (*R*_*i*_, *i* ∈ *U*), where *R*_*i*_ = 1(0) according to the *i*th unit belongs to the population of respondents (non-respondents). In case of missing completely at random (MCAR) non-response factor *R* and the study variable *Y* are uncorrelated and one can ignore the non-response or just apply different imputation techniques [36]. When the stratification variable ***R*** is related to the study variable *Y*, the model for the respondents differs from that of the non-respondents such as in above example the population models may differ among men and women, youngers and elders and deprived and settle areas. To capture this difference, we specify the model of respondents and non-respondents in the population separately according to the values of ***R*** such that

$$\begin{array}{c} Y_{1}=x_{1}\beta_{r}+\epsilon_{1}\;\;\text{for}\;\;R_{i}=1 \end{array}$$ (3)

$$\begin{array}{c} Y_{2}=x_{2}\beta_{nr}+\epsilon_{2}\;\;\text{for}\;\;R_{i}=0\;\;\text{for}\;i\in U, \end{array}$$ (4)

where ***β*_*r*_** and ***β*_*nr*_** are the vectors of regression coefficients corresponding to the respondents the non-respondents respectively. Consequently, we get sub-populations *U*_1_ and *U*_2_ such that *U* = *U*_1_ ∪ *U*_2_, where *U*_1_ and *U*_2_ are the subsets of *U* denoting populations of respondents and non-respondents with sizes *N*_1_ and *N*_2_ respectively. It is assumed that the error terms are independently and identically distributed (IID) with means *E*_*m*_(***ϵ***_1_) = *E*_*m*_(***ϵ***_2_) = 0 with model variances $V_{m}\left(\epsilon_{1}\right)=\sigma_{1}^{2}I_{N1},$ and $V_{m}\left(\epsilon_{2}\right)=\sigma_{2}^{2}I_{N2}$, where $I_{N_{1}}$ and $I_{N_{2}}$ are the identity matrix of order *N*_1_ and *N*_2_ respectively. Separation of model is straight forward when we have exact knowledge about the occurrence of non-response and a related stratification variable which is almost impossible in real world problem. As it is not possible to have such information that separates the underlying model exactly into the respondents and the non-respondents. One way to overcome this problem may be to use two phase sampling for obtaining information on stratification variable. In which we select a larger sample on first phase and observe the stratification variable (i.e. respondents are marked as respondents according to their behavior to respond the first phase survey are observe such factor which cause non-response) and estimate the proportions of units fall in sub-populations i.e. λ_1_ = *N*_1_/*N* and λ_2_ = *N*_2_/*N*. These information then can be used at second phase for estimating population parameters of the study variable. Before going toward our proposal, we discuss the estimation of population total without sub-sampling non-respondents which help us in knowing how the non-response creates biasedness in estimation of total.

### 3.1 Estimation of total without sub-sampling

For a sample *s* of size *n* assume that only *n*_1_ units respond while remaining *n*_2_ units don’t respond. The prediction problem given in Section 2 becomes $T_{y}=W_{s_{1}}^{T}Y_{s_{1}}+W_{{\bar{s}}_{1}}^{T}Y_{{\bar{s}}_{1}}+W_{2}^{T}Y_{2}$, where $W_{s_{1}}^{T}$, $W_{{\bar{s}}_{1}}^{T}$, and $W_{2}^{T}$, are vectors of weights associated with *n*_1_ respondents, *N*_1_ − *n*_1_ non-sampled units from responding population, and *N*_2_ units from non-responding population respectively. Further, $W_{{\bar{s}}_{1}}^{T}Y_{{\bar{s}}_{1}}+W_{2}^{T}Y_{2}$ is unknown and can be predicted using sample at hand and the auxiliary information for the non-responded and non-sampled values. A predictive estimator for population total based on respondents only, can be found as follow:

$$\begin{array}{c} {\hat{T}}_{y1}=W_{s_{1}}^{T}y_{s_{1}}+W_{{\bar{s}}_{1}}^{T}x_{{\bar{s}}_{1}}b_{1}+W_{2}^{T}x_{2}b_{1}, \end{array}$$ (5)

where ***b***_1_ is the vector of OLS estimates of ***β***_1_ based on *n*_1_ respondents. The model bias of ${\hat{T}}_{y1}$ is

$$\begin{array}{c} B_{m}\left({\hat{T}}_{y1}\right)=W_{s_{1}}^{T}x_{2}(\beta_{1}-\beta_{2}). \end{array}$$ (6)

See Appendix A1 for proof. ${\hat{T}}_{y1}$ is unbiased estimate of *T*_*y*_ if the vectors of coefficients for the responding and non-responding sub-populations are same i.e. ***β***_1_ = ***β***_2_, this is equivalent to regression imputation. This situation occurs when Behavior of the responding and the non-responding populations are same allowing us to ignore the non-response just as reduced sample size. We obtain model mean squared error (M-MSE) of the total estimator ${\hat{T}}_{y1}$ as

$$\begin{array}{cc} MSE_{m}\left({\hat{T}}_{y1}\right)= & {\left\{B_{m}\left({\hat{T}}_{y1}\right)\right\}}^{2}+V_{m}\left({\hat{T}}_{y1}\right)\\ = & {\left\{B_{m}\left({\hat{T}}_{y1}\right)\right\}}^{2}+\sigma_{1}^{2}(n_{1}+W_{{\bar{s}}_{1}}^{T}x_{{\bar{s}}_{1}}{\left(H_{s_{1}}\right)}^{-1}x_{{\bar{s}}_{1}}^{T}W_{{\bar{s}}_{1}})\\ & +\sigma_{2}^{2}(W_{2}^{T}x_{2}{\left(H_{s_{1}}\right)}^{-1}x_{2}^{T}W_{2}). \end{array}$$ (7)

The subscript *m* shows that expectation is applied over model. The model-mean squared error (M-MSE) given in (7) depends on random sample under designed-based point of view. Consequently, it varies with sampling fluctuations. To obtain a fix value, we apply expectation with respect to S.D.

### 3.2 Estimation of total with sub-sampling

As we already discussed, there are several approaches for handling the problem of non-response in sample literature. A suitable approach may be chosen according to the type of non-response (full or partial), the accessibility of the auxiliary variable(s) and the validity of the underlying response model for handling the problem. In general, re weighting is used to deal with full (non-availability of units) non-response. Imputation is preferably applied for dealing with partial non-response although it can be applied for full non-response if appropriate auxiliary information is available. Re-weighting eliminates or at least reduces total non-response bias [36, 37]. While the sub-sampling method introduced by [11] provides a good adjustment for non-response bias and yield unbiased estimator for the population mean when the non-response variable *R* is significantly correlated with the survey outcome.

In this study, we develop a model-based estimator for population total by adjusting non-response using sub-sampling procedure. As the models described in (3) and (4) have different parameters it is inevitable to obtain information about both sub-populations. The sample information obtained from respondents alone leads to biased estimate for the population total of the whole population. For estimating the relationship between the study and the auxiliary variables for the population of non-respondents and estimating total, we need some information from non-respondents as well. The sampling mechanism in Section 2 is based on the respondents from first sample which don’t provide any information about the population model of non-respondents. The sub-sampling introduced by [11] is the best alternative to handle such situation of non-response which assumes the mode of data collection on first round was inexpensive and then a more stronger mode of interview is employed for sub-sampling non-respondents. The rationale behind taking a sub-sample instead of following all non-respondent is the fact that taking information from all non-respondents by using stronger mode of interview increases survey cost. Sometime randomized response techniques (more expensive and complex) are applied to gather information on second call [38]. The method assumes sub-sampling ${\overset{´}{n}}_{2}=\frac{n_{2}}{k}$ (*k* > 1) units from *n*_2_ units selected and not respond on first round, using some stronger mode of interview (face to face survey, telephonic survey etc). The estimation process covers two prediction problems (i) predicting *N*_1_ − *n*_1_ non-sampled units from the sample taken from the first round using model given in (3) and (ii) predicting *N*_2_ − *n*_2_ (non-sampled)+ *n*_2_ − ${\overset{´}{n}}_{2}$ (non-responded) units on the basis of sample obtained on second round using the model relationship given in (4). Let ${\overset{´}{s}}_{2}$ be the sub-sample of size ${\overset{´}{n}}_{2}$ selected from *s*_2_ and ${\overset{´}{\bar{s}}}_{2}=U_{2}-{\overset{´}{s}}_{2}$ be the set representing non-sampled values from the population of non-respondents. Now the outcome vector for respondents is further partitioned as $Y_{1}={\left(Y_{s_{1}}:Y_{{\bar{s}}_{1}}\right)}^{T}$ and for non-respondents $Y_{2}={\left(Y_{{\overset{´}{s}}_{2}}:Y_{{\overset{´}{\bar{s}}}_{2}}\right)}^{T}$. The matrix ***x***, the vector ***W*** and the random error vector ***ϵ*** are also partitioned into sampled and non-sampled parts in same way. The population total of the study character is now expressed as $T_{y}=W_{s_{1}}^{T}Y_{s_{1}}+W_{{\bar{s}}_{1}}^{T}Y_{{\bar{s}}_{1}}+W_{{\overset{´}{s}}_{2}}^{T}Y_{{\overset{´}{s}}_{2}}+W_{{\overset{´}{\bar{s}}}_{2}}^{T}Y_{{\overset{´}{\bar{s}}}_{2}}$ after replacing known values of the response units, we have $T_{y}=W_{s_{1}}^{T}y_{s_{1}}+W_{{\bar{s}}_{1}}^{T}Y_{{\bar{s}}_{1}}+W_{{\overset{´}{s}}_{2}}^{T}y_{{\overset{´}{s}}_{2}}+W_{{\overset{´}{\bar{s}}}_{2}}^{T}Y_{{\overset{´}{\bar{s}}}_{2}}$. The problem is to predict $W_{{\bar{s}}_{1}}^{T}Y_{{\bar{s}}_{1}}+W_{{\overset{´}{\bar{s}}}_{2}}^{T}Y_{{\overset{´}{\bar{s}}}_{2}}$. The first part is predicted on the basis of sample obtained on first round along with model given in (3) and the second part is predicted on the basis of sample obtained on second round and the model given in (4). Under the sub-sampling technique a linear unbiased predictor for *T*_*y*_ is

$$\begin{array}{c} {\hat{T}}_{y}^{*}=W_{s_{1}}^{T}y_{s_{1}}+W_{{\bar{s}}_{1}}^{T}x_{{\bar{s}}_{1}}b_{r}+W_{{\overset{´}{s}}_{2}}^{T}y_{{\overset{´}{s}}_{2}}+W_{{\overset{´}{\bar{s}}}_{2}}^{T}x_{{\overset{´}{\bar{s}}}_{2}}b_{2}, \end{array}$$ (8)

where $W_{s_{1}}^{T}$, $W_{{\bar{s}}_{1}}^{T}$, $W_{{\overset{´}{s}}_{2}}^{T}$ and $W_{{\overset{´}{\bar{s}}}_{2}}^{T}$ are the vectors of known weights for the values corresponding to the groups mentioned in subscripts. The estimates of model parameters ***β***_1_ and ***β***_2_ are obtained by solving the normal equations $(H_{s_{1}})b_{1}=x_{s_{1}}^{T}y_{s_{1}}$ and $(H_{{\overset{´}{s}}_{2}})b_{2}=x_{{\overset{´}{s}}_{2}}^{T}y_{{\overset{´}{s}}_{2}}=H_{{\overset{´}{s}}_{2}}$ respectively, where $H_{s_{1}}=x_{s_{1}}^{T}x_{s_{1}}$ and $H_{{\overset{´}{s}}_{2}}=x_{{\overset{´}{s}}_{2}}^{T}x_{{\overset{´}{s}}_{2}}$ are the hessian matrix for the first round sample and sub-sample respectively. The well-known GMT provides the evidence that the OLS estimators are the best linear unbiased estimators (BLUE) of the parameters ***β***_1_ and ***β***_2_ when the observations obtained on first round sample *s*_1_ and the second round sample ${\overset{´}{\bar{s}}}_{2}$ follows two different population models with independently and identically distributed error terms. In designed based point of view, the selection of sub-sample ${\overset{´}{s}}_{2}$ depends on the selection of *s*_1_, hence the assumption of independence is no more valid. To proceed we need the assumption of model independency only. The separation of population as the respondents and the non-respondent is based on the values of *R* which is already discussed in previous section. The role of the variable *R* is same as the role of stratification variable in stratified sampling which is merely used to separate populations into respondents and non-respondents. Hence more correlation between the non-response factor (*R*) and the study variable is a requirement for using the sub-sampling approach. The case of low correlation between the study variable and the non-response variable can be handled through weighting adjustment and imputation techniques discussed in literature review. However the literature of sub-sampling technique reveals that the efficiency of the sub-sampling estimator is not affected by this correlation. But in case of presence of significant correlation proceeding with just respondents on first call may produce invalid and inconsistent statistical inference.

Note that respondents on first sample always represent the responding population *U*_1_. While the non-respondents on first sample may or may not represent the population of the non-respondent *U*_2_ as it depends on the degree of relationship between *R* and *Y* and the nature of occurrence of non-response (whether it is ignorable or not). The model bias of ${\hat{T}}_{y}^{*}$ is derived in Appendix A 2, and given by

$$\begin{array}{c} B_{m}\left({\hat{T}}_{y}^{*}\right)=W_{{\bar{s}}_{1}}^{T}[x_{{\bar{s}}_{1}}\beta_{1}-x_{{\bar{s}}_{1}}\beta_{1}]+W_{{\overset{´}{\bar{s}}}_{2}}^{T}[x_{{\overset{´}{\bar{s}}}_{2}}\beta_{2}-x_{{\overset{´}{\bar{s}}}_{2}}\beta_{2}]=0. \end{array}$$ (9)

${\hat{T}}_{y}^{*}$ is model unbiased if all of the OLS assumptions are satisfied for the populations of the respondents and non-respondents. Assuming unbiasedness model variance of the total estimator under non-response is obtained as

$$\begin{array}{c} V_{m}\left({\hat{T}}_{y}^{*}\right)=n_{1}\sigma_{1}^{2}+{\overset{´}{n}}_{2}\sigma_{2}^{2}+\sigma_{1}^{2}W_{{\bar{s}}_{1}}^{T}x_{{\bar{s}}_{1}}{\left(H_{s_{1}}\right)}^{-1}x_{{\bar{s}}_{1}}^{T}W_{{\bar{s}}_{1}}+\sigma_{2}^{2}W_{{\overset{´}{\bar{s}}}_{2}}^{T}x_{{\overset{´}{\bar{s}}}_{2}}{\left(H_{{\overset{´}{s}}_{2}}\right)}^{-1}x_{{\overset{´}{\bar{s}}}_{2}}^{T}W_{{\overset{´}{\bar{s}}}_{2}} \end{array}$$ (10)

Taking expectation with respect to S.D we get

$$\begin{array}{cc} E_{D}\{V_{m}\left({\hat{T}}_{y}^{*}\right)\}= & N_{1}\sigma_{1}^{2}+N_{2}\sigma_{2}^{2}+E_{D_{1}}[\sigma_{1}^{2}W_{{\bar{s}}_{1}}^{T}x_{{\bar{s}}_{1}}{\left(H_{s_{1}}\right)}^{-1}x_{{\bar{s}}_{1}}^{T}W_{{\bar{s}}_{1}}\\ & +\sigma_{2}^{2}E_{D_{2}}(W_{{\overset{´}{\bar{s}}}_{2}}^{T}x_{{\overset{´}{\bar{s}}}_{2}}{\left(H_{{\overset{´}{s}}_{2}}\right)}^{-1}x_{{\overset{´}{\bar{s}}}_{2}}^{T}W_{{\overset{´}{\bar{s}}}_{2}})], \end{array}$$ (11)

where $E_{D_{1}}$ and $E_{D_{2}}$ are expectations with respect to S.D used for selecting first sample and sub-sample respectively. The first component of the expected model-variance depends on the error variances while the second component depends on the inverse of the matrix *H* = ***x***^*T*^***x*** for the first sample and the sub-sample. Hence, for smaller variance the population units with larger sampled values of all included covariates should be prefer. [39] provided a detail discussion on optimum selection of units under different population models.

## 4 Estimation of total with sub-sampling under super-collinearity and heteroscedasticity

While applying linear regression model for predicting the non-sampled values from the population of non-respondents the number of input variables (regressors) may greatly exceeds the number of observations i.e. ${\overset{´}{n}}_{2}<\left(p+1\right)$ as we are sub-sampling a relatively small portion of non-respondents. In such situations, fitting the full model to the non-respondents without penalization will result in wider prediction intervals, and the normal equations may not have trivial solution as the matrix $H_{{\overset{´}{s}}_{2}}$ does not possess the full rank property. It is not possible to estimate the parameters of the model when $H_{{\overset{´}{s}}_{2}}$ is singular i.e. not of full rank. This situation is called super-collinearity or ill-conditioning. The problem of super-collinearity can be solved using ridge regression. To get an estimate for ***β***_2_, when there is super-collinearity in ***x***_2_, we use ad-hoc fix method proposed by [40] for resolving singularity of $H_{{\overset{´}{s}}_{2}}$. We simply replace $H=H_{{\overset{´}{s}}_{2}}$ by $H\left(v\right)=H_{{\overset{´}{s}}_{2}}+vI_{p+1}$ with *v* ∈ [0, ∞]. The scalar *v* is called tuning parameter or penalty parameter. A clearly defined estimator for *β*_2_ obtained even for high-dimensional data matrix $\left({\overset{´}{n}}_{2}\leq p\right)$ for a strictly positive *v* is $b_{2}\left(v\right)=H{\left(v\right)}^{-1}x_{{\overset{´}{s}}_{2}}^{T}y_{{\overset{´}{s}}_{2}}$. Using *b*_2_(*v*) in (8), we obtain a partially ridge regression (PRR) estimator (as the concept of ridge regression is used for non-responding part only) for population total which is given by

$$\begin{array}{c} {\hat{T}}_{y}^{*}=W_{s_{1}}^{T}y_{s_{1}}+W_{{\bar{s}}_{1}}^{T}x_{{\bar{s}}_{1}}b_{1}+W_{{\overset{´}{s}}_{2}}^{T}y_{{\overset{´}{s}}_{2}}+W_{{\overset{´}{\bar{s}}}_{2}}^{T}x_{{\overset{´}{\bar{s}}}_{2}}b_{2}\left(v\right). \end{array}$$ (12)

The expressions for model-bias and expected model-MSE of the PRR estimator of the total in presence of non-response are obtained by replacing *H*(*v*) by *H* in (10) and (11). Following [41] a range for *v* in which the model-MSE of $x_{{\overset{´}{\bar{s}}}_{2}}b_{2}\left(v\right)$ is smaller than the model-variance of $x_{{\overset{´}{\bar{s}}}_{2}}b_{2}$ is

$$\begin{array}{c} 0<v<\frac{2}{\left[-min\left(0,\psi_{2}\right)\right]}, \end{array}$$ (13)

where *ψ*_2_ is the minimum eigen-value of the matrix ${\left(H_{{\overset{´}{s}}_{2}}\right)}^{-1}-\frac{\beta_{2}\beta_{2}^{T}}{\sigma_{2}^{2}}$. PRR is also applicable for predicting non-sampled respondents when *n*_1_ < (*p* + 1) leading to super-collinearity in the respondents.

Another major problem that arises in estimation of so called superpopulation parameters is the violation of assumption of homoscedasticity is violated. In presence of heteroscedasticity one has

$$\begin{array}{c} V_{m}(Y_{1}|x_{1})=\sigma_{1}^{2}V_{1}\;\;\text{for}\;\;R=1 \end{array}$$ (14)

$$\begin{array}{c} V_{m}(Y_{2}|x_{2})=\sigma_{2}^{2}V_{2}\;\;\text{for}\;\;R=0, \end{array}$$ (15)

where ***V***_1_ = *diag*(*V*_1*ii*_,*i* ∈ *U*_1_) and ***V***_2_ = *diag*(*V*_2*ii*_, *i* ∈ *U*_2_) units specific variances for respondents and non-respondents respectively. Here *V*_1*ii*_ = *V*_*m*_(*Y*_1*i*_|***x***_1*i*_) = *υ*(***x***_1*i*_) and *V*_2*ii*_ = *V*_*m*_(*Y*_2*i*_|***x***_2*i*_) = *υ*(*x*_2*i*_), where ***x***_1*i*_ and ***x***_2*i*_ are the vectors of the auxiliary variables corresponding to the *i*th unit in respondents and non-respondents respectively. In such situations, OLS estimators for the regression coefficients may have higher variances. If we have information about the variance structure for the populations of respondents and non-respondents (assuming zero correlation between the units), we can adopt weighted least square (WLS) method of estimation. The WLS estimators of ***β***_1_ and ***β***_2_ are $b_{1wls}={\left(x_{s_{1}}^{T}V_{s_{1}}^{-1}x_{s_{1}}\right)}^{-1}x_{s_{1}}^{T}V_{s_{1}}^{-1}y_{s_{1}}$ and $b_{2wls}=(x_{{\overset{´}{s}}_{2}}^{T}V_{{\overset{´}{s}}_{2}}^{-1}x_{{\overset{´}{s}}_{2}})x_{{\overset{´}{s}}_{2}}^{T}V_{{\overset{´}{s}}_{2}}^{-1}y_{{\overset{´}{s}}_{2}}$ respectively, where $V_{1}=\left(V_{s_{1}},V_{{\bar{s}}_{1}}\right)$

$$\begin{array}{c} V_{1}=[\begin{array}{cc} V_{s_{1}} & 0\\ 0 & V_{{\bar{s}}_{1}} \end{array}]\;\;\text{and}\;\;\;V_{2}=[\begin{array}{cc} V_{{\overset{´}{s}}_{2}} & 0\\ 0 & V_{{\overset{´}{\bar{s}}}_{2}} \end{array}]. \end{array}$$

The sub-matrices are also diagonal assuming zero correlation between the error terms corresponding to the respobndents and the non-respondents. A WLS estimator for *T*_*y*_ in presence of non-response is obtained by replacing ***b***_1*wls*_ and ***b***_2*wls*_ by ***b***_1_ and ***b***_2_ respectively in (8). It is assumed that the variance structures of the responding and non-responding population are known and depend on covariates whose values are known for each population unit. In practice, for many types of data set, the structure of weights (inverse of variance) is usually unknown, so one has to perform an ordinary least squares (OLS) regression first to estimate the variance structure and obtain estimates for the population regression coefficients after performing an iterative process which is commonly known as generalized least square (GLS).

## 5 Application

**Fig 1:**
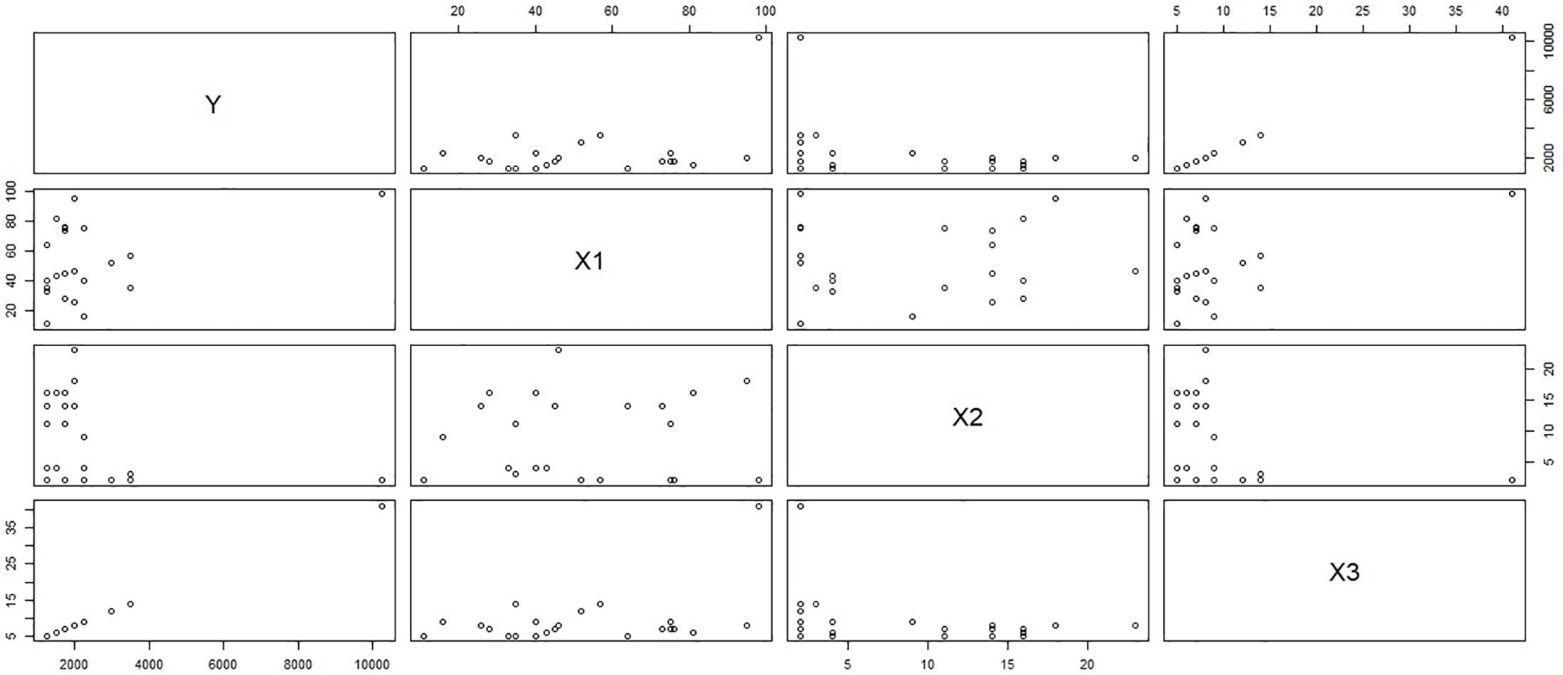
Behavior of non-respondents with λ_2_ = 0.4.

**Fig 2:**
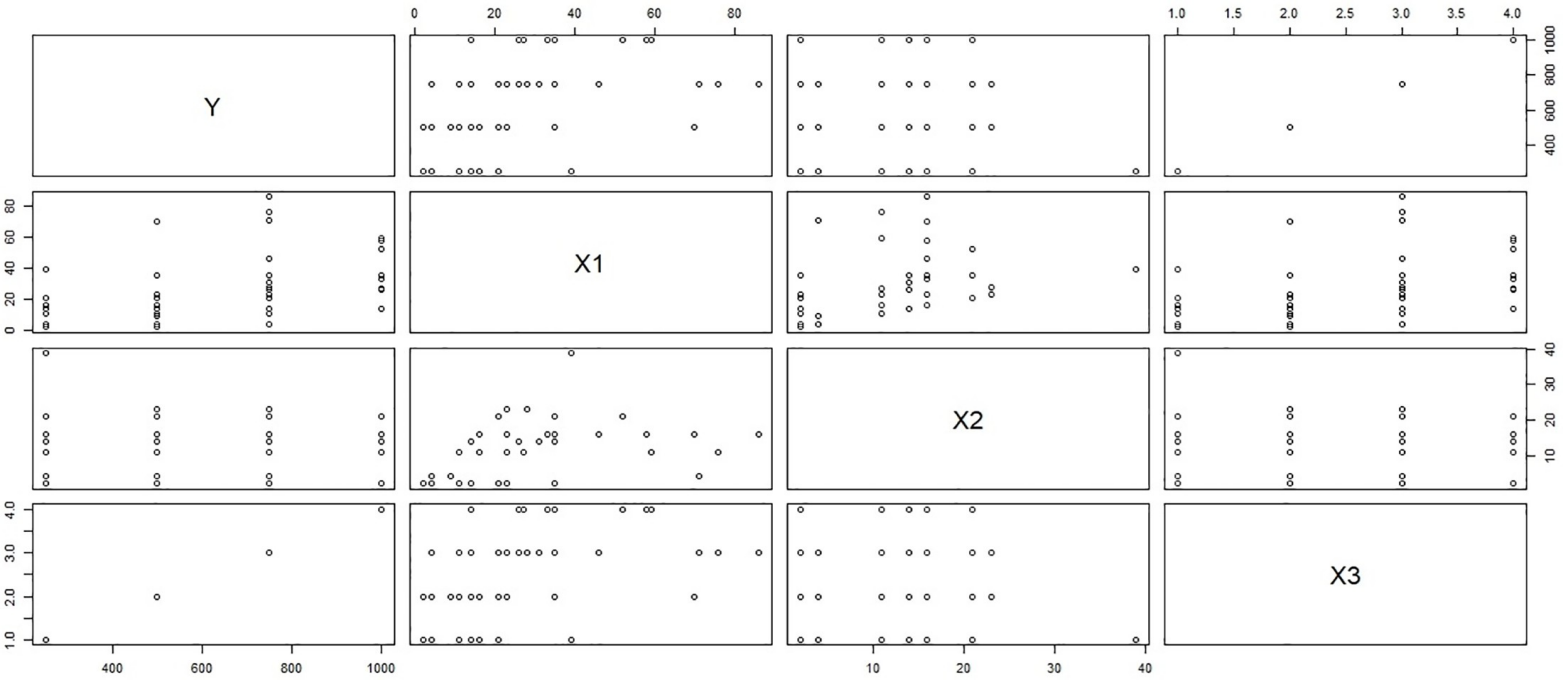
Behavior of respondents with λ_2_ = 0.4.

**Fig 3:**
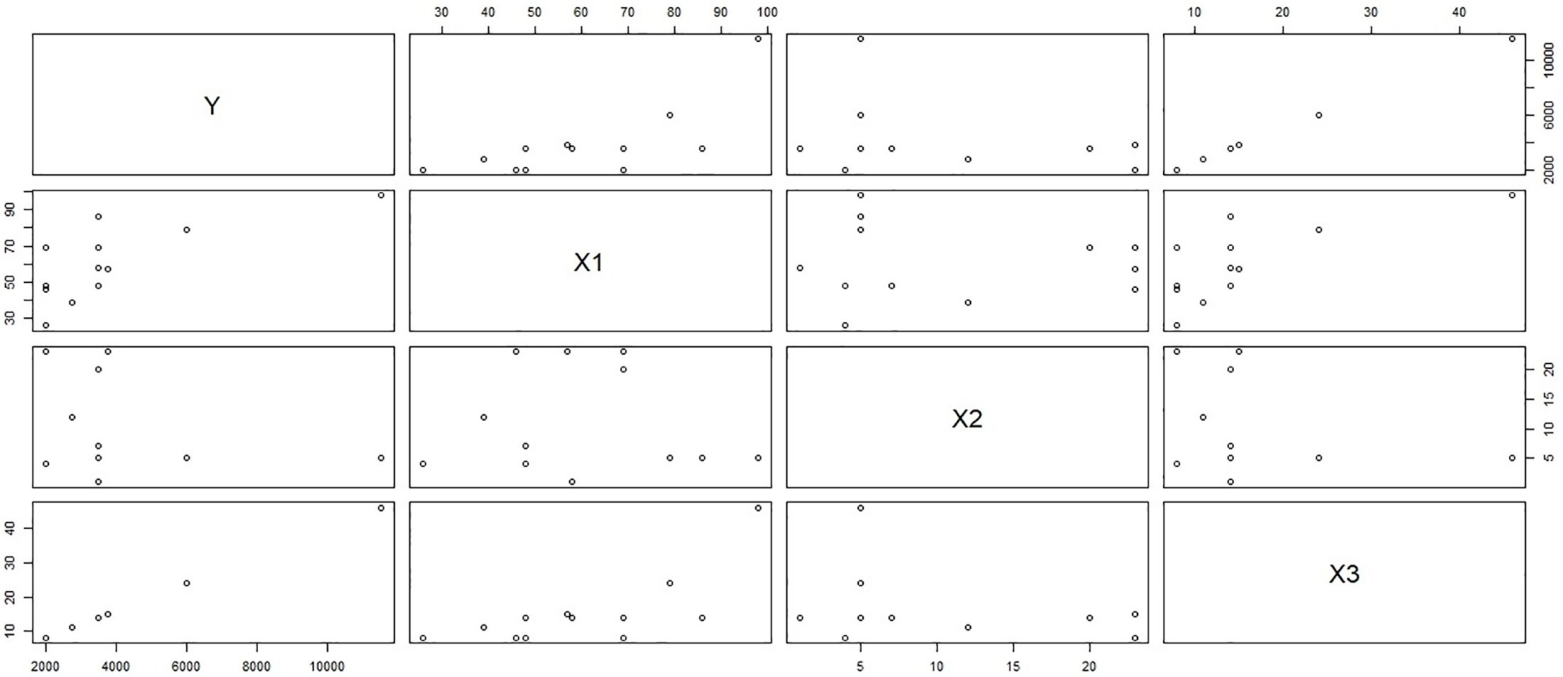
Behavior of non-respondents with λ_2_ = 0.2.

**Fig 4:**
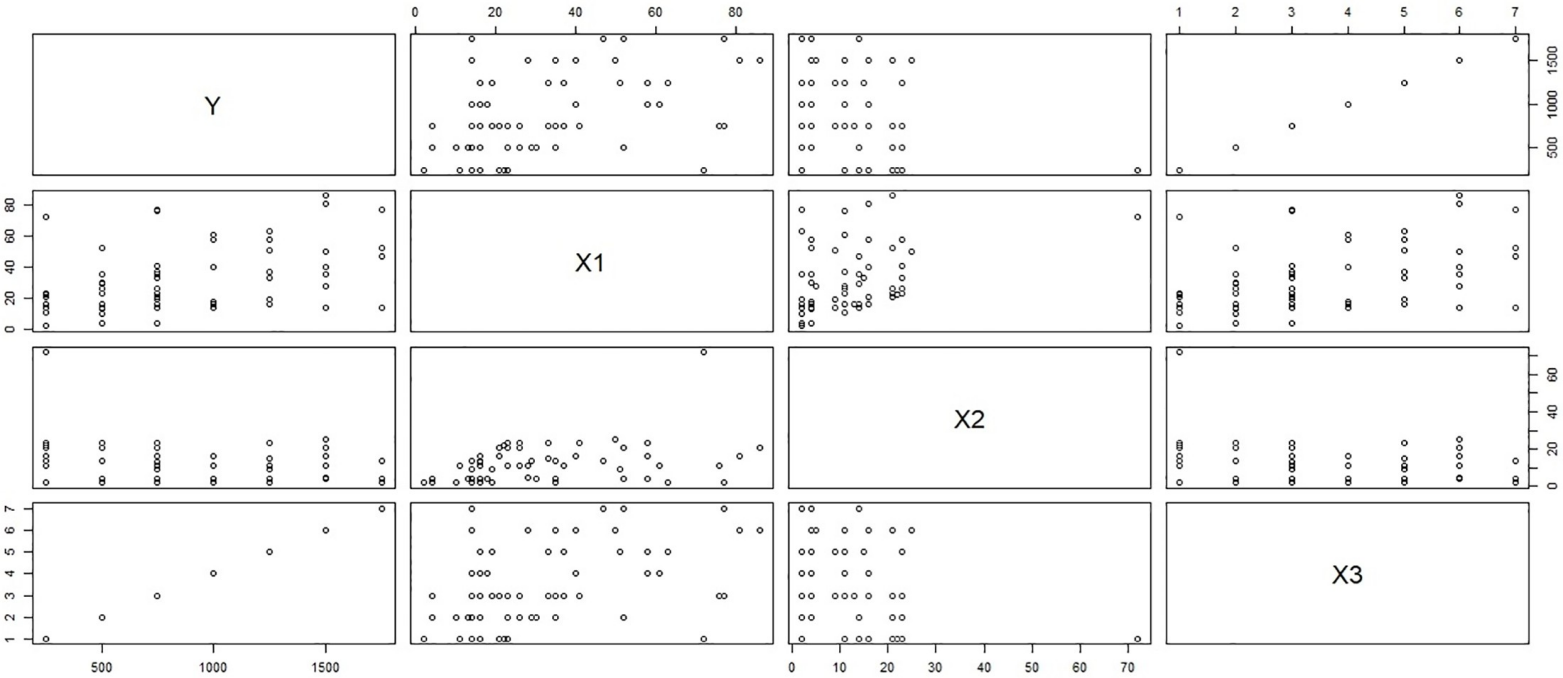
Behavior of respondents with λ_2_ = 0.2.

A real data set taken from [42] is applied to investigate the behavior of our proposed model-based estimator. The data set is given as supporting information S1 Data 7 to this paper. The data consist of 748 blood donors on following variables:

***y*** = Monetary total blood donated in c.c., ***x***_1_ = Time (months since first donation), ***x***_2_ = Recency (months since last donation) and ***x***_3_ = Frequency (total number of donation). Considering the above 748 blood donors as our population of interest, we select a sample of size 100 using simple random sampling without replacement. The scatter plot matrix between the variables in the sample selected on first call and the sub-sample collected on the second call represents the relationship between the variables in the population of respondents and non-respondents for response rates λ_2_ = 0.4 (Figs 1 and 2) and λ_2_ = 0.4 (Figs 3 and 4). Fig 1 shows the relation between the study variable *y* and the predictors *x*_1_, *x*_2_, and *x*_3_ for the sampled respondents which shows that the study variable *y*, is highly related to *x*_3_ and moderately related to *x*_1_ but weakly related to *x*_2_. Fig 2 portrays the relationship between variables for the sub-sampled non-respondents which is different from the relationship in Fig 1 which shows the relevancy of the data to our proposed sampling mechanism. One can observe the similar relationship between the variables for λ = 0.2 from upper triangle of Figs 3 and 4. Hence our proposal works here as the relationship between the total monetary blood donated and its three determinants have different relationship for the population of the respondents and the sub-population of the non-respondents which is the main assumption of our data collection mechanism. We select half (*k* = 2) of the non-respondent selected on first call for sub-sampling on second call.

Further to see the magnitude of the prediction error, we provide a bootstrap sampling procedure taking different non-response rate (say λ_2_) in the population. We generate a new variable *R* associated with each 748 cases which posses value 1 if the *i*th unit has an outcome greater than the λ_2_ th percentile of all the *y* values in the data set otherwise zero.

1. A sample of size *n* (for *n* = 100, 200) is taken from the data using simple random sampling without replacement and divide it them into the respondents and the non-respondents according to the value of *R* and observe *n*_1_ and *n*_2_.
2. Select a sub-sample of size ${\overset{´}{n}}_{2}=\frac{n_{2}}{k}$ (taking *k* = 2,4) from *n*_2_ non-respondents again using simple random sampling without replacement and compute the estimator using information obtain from first and second samples. We take *p* = 2 to avoid the problem of super-collinearity in our situation.
3. Repeat Step 2, 2000 times to get expected value from the sub-sampling. The sub-sampling does not alter results of ${\hat{T}}_{1}$ as it is based on sample from respondents only.
4. Repeat Steps 1-3, 5000 times to obtain a stable value of prediction variance and bias for both estimators.

The prediction bias and variances are computed as follows:

$$\begin{array}{c} RB({\hat{T}}_{y1})=E_{D1}E_{D2}[\frac{{\hat{T}}_{y1}-T_{y}}{T_{y}}] \end{array}$$ (16)

$$\begin{array}{c} RMSE({\hat{T}}_{y1})=E_{D1}E_{D2}{\left[\frac{{\hat{T}}_{y1}-T_{y}}{T_{y}}\right]}^{2} \end{array}$$ (17)

The RB and RMSE for the [11]-type estimator for the population total are obtained by replacing ${\hat{T}}_{y1}$ by ${\hat{T}}_{y}^{*}$ in Eqs (16) and (17) respectively. Table 1 provides relative bias (RB) and relative mean squared error (RMSE) of the total estimator based on the sample on first call for different combinations of *n*, λ_2_ and *k*.

**Table 1:** Relative bias and MSE.

*n*	*k*	$RB({\hat{T}}_{y1})$	$RB({\hat{T}}_{y}^{*})$	$RMSE({\hat{T}}_{y1})$	$RMSE({\hat{T}}_{y}^{*})$
λ_2_ = 0.5					
100	2	-0.56463	0.03325	0.32017	0.01014
	4		0.01626		0.02009
200	2	-0.56646	0.04592	0.32130	0.00575
	4		0.03085		0.00996
λ_2_ = 0.25					
100	2	-0.36311	0.01615	0.13352	0.00766
	4		-0.00332		0.02661
200	2	-0.36719	0.02492	0.13554	0.00371
	4		0.01449		0.00732
λ_2_ = 0.10					
100	2	-0.19545	-0.00931	0.04023	0.42009
	4		-0.02266		0.47775
200	2	-0.19806	-0.00607	0.04005	0.03924
	4		-0.00239		0.04046

The results in Table 1 are reported assuming non-response rate λ_2_ at 50%, 25%, and 10%. RB of both estimators go to zero as non-response rate falls toward zero which assures that for full response it vanishes while the sub-sampling method produce ignorable bias as compared to direct method which is the attractive feature of this method. Further from Table 1, one can observe that RMSE is smaller in case of sub-sampling non-respondents, i.e. taking interview of additional non-respondents through some stronger mode of interview, for every choices of λ_2_. RB and RMSE of ${\hat{T}}_{y}^{*}$ tend to increase with decrease in non-response rate in the population which shows that our proposed technique works well for higher non-response rates as compared to lower smaller ones. RB and RMSE of the model based total estimator go down while increasing sub-sample size ${\overset{´}{n}}_{2}$ (decreasing *k*) as expected. Further, this error decreases when population has smaller non-response rate λ_2_. In upcoming section, we provide a simulation study to provide a detailed picture of the performance of estimators in terms of design bias and mean squared error.

## 6 Simulation study

To see the long run behavior of the proposed estimators in terms of bias and efficiency, a simulation study, generating a hypothetical population, is conducted. Following [43], a matrix ***z*** = (*z*_*ij*_, *i* = 1, 2, 3, …, *N*, *j* = 1, 2, …, *p*) with *p* variate each generated from *N*(100,1), has been constructed with *N* = 10,000 observations. The *ij*th element of the auxiliary matrix ***x*** is computed as *x*_*ij*_ = (1 − *ρ*)^0.5^ × *z*_*ij*_ + *ρ* × *z*_*ij*_, where *ρ* is the degree of linear relationship between ***x*** and ***z*** to be fixed in advance. The vector of the study variable (***y***) is then obtained by using the relationship *y* = ***x****γ* + ***ϵ***, where *γ* is the vector of coefficients which are computed as the averaged eigen vectors corresponding to the eigen values of *H* = ***x***^*T*^***x*** that are greater than unity and ***ϵ*** ∼ *N*(0, *σ*^2^***I***_*N*_) is randomly generated error term. It is assumed that the variance is of homoscedastic nature with constant diagonal *σ*^2^. We fix *σ*^2^ at 0.01, 0.1 and 1. The data consist of (***y***, **1**_*N*_, ***x***, *R*_*i*_), where **1**_*N*_ is the vector of 1’s. *R*_*i*_ takes value 1 if the *i*th value of variable *y* falls in a threshold lower than (1 − λ_2_)th quantile in the population, where λ_2_ is non-response rate in the population. In real life, we suggest to choose *R* in form of some observable covariates or latent variables. The simulation study is conducted in following three steps.

- Take a random sample of size *n* from the population generated through the mechanism described above and split it into *n*_1_ respondents and *n*_2_ non-respondents according to the values of *R*_*i*_.
- Select a sub-sample of size ${\overset{´}{n}}_{2}$ from *n*_2_ non-respondents for fix *k*.
- Estimate the population total (*T*_*y*_) using estimated models from samples obtained on Steps 1 and 2.
- Simulate Steps 2–3 500 times and average the values of estimates.
- Repeat Steps 1–4 2000 times to obtain prediction errors to obtain 2000 estimated values.

The bias (B) and mean squared error (MSE) of the proposed total estimators are computed using the formula given in Eqs (16) and (17) respectively after removing the denominators as the generated values are already standardized. The subscript *v* is used for the results where prediction is performed using PRR.

Tables 2–4 provide the bias of the PPR estimator and mean squared error of both estimators for different combinations of *σ*^2^, λ_2_, *ρ*, *n* and *k* in nested order. We obtain results for *p* = 5 and *p* = 8 but the result for *p* = 5 is not reported here for the sake of space. Tables 2, 3 and 4 provide the prediction error measures (B and MSE) for *σ*^2^ = 0.01, *σ*^2^ = 0.1 and *σ*^2^ = 1 respectively. From Tables 2–4, one can see that the bias of the PRR total estimator tends to increase with increase in *k*. This implies selecting a smaller sub-sample increases the bias in estimation due to sampling error although this bias depends on the magnitude of the tuning parameter *v*. MSE of the total estimator under multiple regression and PRR both increase with increase in *k* which shows that MSE of the estimators grows with smaller sub-samples from non-respondents. The PRR total estimator is more sensitive to the change in *k*, in terms of MSE, as the optimum value of the tuning parameter *v* is estimated from sub–sample. In practice *v* might be computed using data available from previous surveys of the same population or through expert judgment. The estimation methods of *v* by minimization of prediction error are available in [43]. Moreover, whatever model we use for prediction, the MSE values of the total estimators depend on the sample size of respondents and sub-sample of non-respondents. The simulated results are provided for sample size 100, 150 and 250 with sub-sample size inversely proportional to *k* = 1.5, *k* = 2 and *k* = 3. It can be noticed that MSE values are increasing with increase in *k*. Comparing two portions of Tables 2–4, we observe that the MSE of proposed estimators fall when non-response rate increases which conflicts the efficiency property of the [11] estimator. The reason is the use of separate models and increasing λ_2_ from 0.2 to 0.4 implies(i.e. we are using Model (2) for 40% of the data) which is the main contribution of our proposal in terms of increased precision. Apart from the design parameters, the data generating process also effects the efficiency of the total estimator which can be seen from three different column-panels (for three different choices of the parameter *ρ*) assuming that the correlation between the variables *X* and *Z* are same for all choices of *j* of Tables 2–4.

**Table 2:** Bias and MSEs with *σ*^2^ = 0.01 and *p* = 8.

			*ρ* = 0.5			*ρ* = 0.7			*ρ* = 0.9		
λ_2_	*n*	*k*	$Bias({\hat{T}}_{yv}^{*})$	$MSE({\hat{T}}_{y}^{*})$	$MSE({\hat{T}}_{yv}^{*})$	$Bias({\hat{T}}_{yv}^{*}$	$MSE({\hat{T}}_{y}^{*})$	$MSE({\hat{T}}_{yv}^{*})$	$Bias({\hat{T}}_{yv)}^{*}$	$MSE({\hat{T}}_{y}^{*})$	$MSE({\hat{T}}_{yv}^{*})$
0.2	100	1.5	-1.9045	2632.5429	3410.3629	0.1861	2501.6722	3101.5096	-3.5464	3615.4525	4146.0974
		2	-3.0774	15332.5627	18264.6392	-0.9490	14016.4277	16670.4440	0.8504	22387.5100	24270.7100
		3	-4.7323	63897.5500	67396.2853	-6.8877	17623.5750	18316.7489	-6.1958	102069.3970	105702.5531
	150	1.5	-0.1320	182.0770	181.8869	-0.0860	177.5572	177.5734	-0.0015	0.0115	0.0004
		2	-0.1403	237.1414	238.3950	-0.2664	233.9038	233.6031	0.8081	200.9532	283.5728
		3	0.1429	998.3503	1041.6369	-4.7654	11861.0556	15667.4757	-4.5923	12698.1924	14579.8670
	200	1.5	-0.2404	124.6444	124.7199	0.0072	0.0472	0.1311	-0.0021	0.0776	0.0007
		2	-0.2501	141.0534	140.9511	0.0491	13.0456	30.5921	-0.0023	0.0682	0.0006
		3	-0.2724	225.3042	230.9373	-2.1225	756.4574	1453.1401	-1.2813	556.3236	888.3302
0.2	100	1.5	-0.0017	0.1004	0.0005	-0.5134	309.4638	309.6424	-0.0082	285.7515	286.0365
		2	-0.1018	2.0960	16.3657	-0.5683	399.1314	399.0764	-0.3330	368.5770	369.1162
		3	-1.3227	1081.2742	5169.5272	-0.1887	703.9439	703.9108	-0.3322	681.8513	686.7257
	150	1.5	-0.0010	0.0189	0.0002	-0.3027	176.2154	176.2077	-0.3701	169.2124	169.2489
		2	-0.0015	0.0676	0.0003	-0.2734	222.5153	222.6088	-0.1682	203.0450	203.0715
		3	-0.0023	0.4645	0.0010	-0.5970	336.7227	337.0286	-0.7045	315.0578	314.8267
	200	1.5	-0.0003	0.0057	0.0001	-0.0077	136.3723	136.3636	-0.1989	124.4898	124.5128
		2	-0.0010	0.0167	0.0002	0.0665	166.4728	166.5163	-0.1507	151.5523	151.5717
		3	-0.0016	0.1027	0.0004	0.3372	237.1700	237.1535	-0.0540	217.1780	217.2875

**Table 3:** Bias and MSEs with *σ*^2^ = 0.1 and *p* = 8.

			*ρ* = 0.5			*ρ* = 0.7			*ρ* = 0.9		
λ_2_	*n*	*k*	$Bias({\hat{T}}_{yv}^{*})$	$MSE({\hat{T}}_{y}^{*})$	$MSE({\hat{T}}_{yv}^{*})$	$Bias({\hat{T}}_{yv}^{*}$	$MSE({\hat{T}}_{y}^{*})$	$MSE({\hat{T}}_{yv}^{*})$	$Bias({\hat{T}}_{yv)}^{*}$	$MSE({\hat{T}}_{y}^{*})$	$MSE({\hat{T}}_{yv}^{*})$
0.2	100	1.5	0.0527	761.5122	6.0175	-0.1304	712.9594	32.4517	0.1087	779.5891	101.4044
		2	0.3897	3072.4058	597.1933	-0.1384	2957.2573	884.0712	0.3682	3096.5782	960.3027
		3	-4.1239	18395.7554	12510.7762	-2.1398	18856.2093	13943.7068	0.4578	17867.0923	16000.5730
	150	1.5	-0.0018	102.4919	0.0004	-0.0013	93.5663	0.0004	-0.0018	98.2044	0.0004
		2	-0.0031	320.7221	0.0007	-0.0021	307.4636	0.0007	-0.0031	312.4716	0.0007
		3	-0.0853	2119.3799	393.5367	-0.1952	1936.9254	120.3755	0.2440	2012.8939	584.2767
	200	1.5	-0.0010	31.2990	0.0002	-0.0007	27.9423	0.0003	-0.0006	30.3235	0.0003
		2	-0.0019	103.3161	0.0003	-0.0014	83.5846	0.0003	-0.0012	96.0962	0.0003
		3	-0.0032	501.3613	0.0008	-0.0027	543.5329	0.0007	-0.0024	486.4563	0.0008
0.4	100	1.5	-0.0012	34782.8600	0.0002	-0.0024	35656.4700	0.0002	-0.0013	36537.1700	0.0002
		2	-0.0022	76289.3900	0.0003	-0.0031	73245.7900	0.0003	-0.0032	335.3555	0.0008
		3	-0.0042	202559.5000	0.0012	-0.0052	184955.9000	0.0009	-0.0069	1811.6840	0.0031
	150	1.5	-0.0055	272320.5567	0.0016	-0.0064	247252.1500	0.0012	-0.0094	-21830.7495	0.0043
		2	-0.0070	356208.8767	0.0021	-0.0078	321901.8650	0.0015	-0.0122	-39193.4925	0.0058
		3	-0.0085	440097.1967	0.0027	-0.0092	396551.5800	0.0019	-0.0150	-56556.2355	0.0073
	200	1.5	-0.0100	523985.5167	0.0032	-0.0106	471201.2950	0.0022	-0.0178	-73918.9785	0.0087
		2	-0.0115	607873.8367	0.0037	-0.0120	545851.0100	0.0026	-0.0207	-91281.7215	0.0102
		3	-0.0130	691762.1567	0.0043	-0.0134	620500.7250	0.0029	-0.0235	-108644.4645	0.0117

**Table 4:** Bias and MSEs with *σ*^2^ = 1 and *p* = 8.

λ_2_	*n*	*k*	$Bias({\hat{T}}_{yv}^{*})$	$MSE({\hat{T}}_{y}^{*})$	$MSE({\hat{T}}_{yv}^{*})$	$Bias({\hat{T}}_{yv}^{*}$	$MSE({\hat{T}}_{y}^{*})$	$MSE({\hat{T}}_{yv}^{*})$	$Bias({\hat{T}}_{yv)}^{*}$	$MSE({\hat{T}}_{y}^{*})$	$MSE({\hat{T}}_{yv}^{*})$
0.2		1.5	-0.92480	61377.38088	2732.37605	-1.18547	64137.80092	2116.32394	-1.18547	64137.80092	2116.32394
	100	2	-2.94078	98309.64742	27170.99000	-8.24624	98661.25065	25524.12997	-8.24624	98661.25065	25524.12991
		3	-27.35530	167858.93049	198667.30000	-23.36264	156272.98600	200505.09000	-23.36264	156272.98600	200505.09930
		1.5	-0.00095	32731.19000	0.00010	-0.00101	31548.83000	0.00007	-0.00101	31548.83000	0.00007
	150	2	-0.00206	53798.86000	0.00057	0.66527	50745.12235	273.95232	0.66527	50745.12235	273.95232
		3	-5.45773	98828.28093	18837.24500	-5.31764	95632.41642	20115.99933	-5.31764	95632.41642	20115.99933
		1.5	-0.00050	17985.20000	0.00003	-0.00086	18345.49000	0.00003	-0.00086	18345.49000	0.00003
	200	2	-0.00073	30170.24000	0.00007	-0.00107	32707.66000	0.00007	-0.00107	32707.66000	0.00007
		3	-0.87860	62423.15344	236.95346	-0.58426	63258.35576	189.24416	-0.58426	63258.35576	189.24416
0.4	100	1.5	-0.00054	74174.77000	0.00005	-0.00054	73609.55000	0.00008	-0.26906	296990.10000	186.24990
		2	-0.00123	137269.40000	0.00018	-0.00114	136571.00000	0.00019	-0.00118	142043.00000	0.00012
		3	0.21696	304271.30000	1655.51200	-0.93803	292194.00000	877.38890	-0.26906	296990.10000	186.24990
	150	1.5	-0.00016	27143.28000	0.00002	-0.00049	27164.02000	0.00002	-0.00094	133416.30000	0.00011
		2	-0.00034	54688.47000	0.00004	-0.00062	57434.25000	0.00004	-0.00060	55799.30000	0.00004
		3	-0.00097	138008.00000	0.00014	-0.00116	144321.40000	0.00014	-0.00094	133416.30000	0.00011
	200	1.5	-0.00019	13010.95000	0.00001	-0.00030	12975.12000	0.00001	-0.00070	72677.00000	0.00005
		2	-0.00021	27447.29000	0.00002	-0.00040	27325.85000	0.00002	-0.00039	27794.85000	0.00002
		3	-0.00058	73274.17000	0.00005	-0.00077	75598.08000	0.00006	-0.00070	72677.00000	0.00005

## 7 Conclusion

This article is concerned with utilization of model relationship between the outcome variable and one or more covariate(s) for efficient estimation of population total of the outcome variable in surveys with non-ignorable non-response. A model based version of [11] sub-sampling technique is suggested which assumes that the responding and non-responding population have different models. This assumption may hold for majority of real world situations where the occurrence of non-response is observable like a stratification variable. In public health surveys the non-response occurrence is based on the gender, ethical affiliation, age and other demographic factors of the respondents. In such situations, respondents and non-respondents may have different models. The method assumes that a stratification variable is available to divide the population into respondents and non-respondents which is difficult to obtain in most of real surveys although a two phase sampling method can provide a better stratification variable to divide the population into respondents and non-respondents. It is shown that under linear population model (linear in parameter as well as in variables) the total estimator with sub-sampling is model-unbiased and has smaller model-variance as compared to predictive estimator based on sampled respondents only. The linearity assumption emphasizes on linear in parameters but not restricted to the linearity in variable. Polynomial regression models are also useful for handling non-response in demographic surveys using age as the predictor. The problem of non-response can be well handled using polynomial regression models which is an open area to work in future. While sub-sampling non-respondents the number of observations may become smaller than the number of regressors included in the model leading to problem of super-collinearity. To cope with super-collinearity problem, we suggest a version of ridge regression named, called PRR, for predicting the non-sampled non-respondents. WLS and GLS are suggested for obtaining estimates of the regression coefficients for respondents and non-respondents when error terms for at least one model is of heteroscedasticity nature. To confirm mathematical expressions a numerical study with blood transfusion data has been carried out. The suggested method is applicable to telephonic or web household surveys where households are first contacted with email or telephone call and then non-respondents are followed via face to face surveys where it seems logical to select a sub-sample of non-respondents through more expensive mode (face to face).

### APPENDIX A1. Derivation of bias and MSE ${\hat{T}}_{y1}$ without sub-sampling

$$\begin{array}{cc} B_{m}\left({\hat{T}}_{y1}\right)= & E_{m}({\hat{T}}_{y1}-T_{y})=E_{m}(W_{s_{1}}^{T}y_{s_{1}}+W_{{\bar{s}}_{1}}^{T}x_{{\bar{s}}_{1}}b_{1}+W_{2}^{T}x_{2}b_{1}-T_{y})\\ = & E_{m}(W_{s_{1}}^{T}y_{s_{1}}+W_{{\bar{s}}_{1}}^{T}x_{{\bar{s}}_{1}}b_{1}+W_{2}^{T}x_{2}b_{1}-T_{y})\\ = & E_{m}(W_{{\bar{s}}_{1}}^{T}x_{{\bar{s}}_{1}}b_{1}+W_{2}^{T}x_{2}b_{1}-W_{{\bar{s}}_{1}}^{T}Y_{{\bar{s}}_{1}}-W_{2}^{T}Y_{2})\\ = & E_{m}(Ab_{1}-W_{{\bar{s}}_{1}}^{T}Y_{{\bar{s}}_{1}}-W_{2}^{T}Y_{2})\\ = & E_{m}[A{\left(H_{s_{1}}\right)}^{-1}x_{s_{1}}^{T}y_{s_{1}}-W_{{\bar{s}}_{1}}^{T}Y_{{\bar{s}}_{1}}-W_{2}^{T}Y_{2}]\\ = & A{\left(H_{s_{1}}\right)}^{-1}x_{s_{1}}^{T}E_{m}\left(y_{s_{1}}|x_{s_{1}}\right)-W_{{\bar{s}}_{1}}^{T}E_{m}\left(Y_{{\bar{s}}_{1}}|x_{{\bar{s}}_{1}}\right)-W_{2}^{T}E_{m}\left(Y_{2}|x_{2}\right)\\ = & A{\left(H_{s_{1}}\right)}^{-1}H_{s_{1}}\beta_{1}-W_{{\bar{s}}_{1}}^{T}x_{{\bar{s}}_{1}}^{T}\beta_{1}-W_{2}^{T}x_{2}^{T}\beta_{2}\\ = & A{\left(H_{s_{1}}\right)}^{-1}H_{s_{1}}\beta_{1}-W_{{\bar{s}}_{1}}^{T}x_{{\bar{s}}_{1}}^{T}\beta_{1}-W_{2}^{T}x_{2}^{T}\beta_{2}\\ B_{m}\left({\hat{T}}_{y1}\right)= & W_{2}^{T}x_{2}(\beta_{1}-\beta_{2}) \end{array}$$

where $A=W_{{\bar{s}}_{1}}^{T}x_{{\bar{s}}_{1}}+W_{2}^{T}x_{2}$. The model variance of ${\hat{T}}_{y1}$ is derived as

$$\begin{array}{c} V_{m}\left({\hat{T}}_{y1}\right)=V_{m}(W_{s_{1}}^{T}y_{s_{1}}+W_{{\bar{s}}_{1}}^{T}x_{{\bar{s}}_{1}}b_{1}+W_{2}^{T}x_{2}b_{1}). \end{array}$$

Under OLS assumptions, we have $V_{m}\left(b_{1}\right)=\sigma_{1}^{2}{\left(H_{s_{1}}\right)}^{-1}$. Inserting this result, we get

$$\begin{array}{cc} V_{m}\left({\hat{T}}_{y1}\right)= & \sigma_{1}^{2}(n_{1}+W_{{\bar{s}}_{1}}^{T}x_{{\bar{s}}_{1}}{\left(H_{s_{1}}\right)}^{-1}x_{{\bar{s}}_{1}}^{T}W_{{\bar{s}}_{1}})+\sigma_{2}^{2}(W_{2}^{T}x_{2}{\left(H_{s_{1}}\right)}^{-1}x_{2}^{T}W_{2}). \end{array}$$

The MSE of ${\hat{T}}_{y1}$, is given by

$$\begin{array}{cc} MSE_{m}\left({\hat{T}}_{y1}\right)= & {\left\{B_{m}\left({\hat{T}}_{y1}\right)\right\}}^{2}+V_{m}\left({\hat{T}}_{y1}\right)\\ = & {\left\{B_{m}\left({\hat{T}}_{y1}\right)\right\}}^{2}+\sigma_{1}^{2}(n_{1}+W_{{\bar{s}}_{1}}^{T}x_{{\bar{s}}_{1}}{\left(H_{s_{1}}\right)}^{-1}x_{{\bar{s}}_{1}}^{T}W_{{\bar{s}}_{1}})\\ & +\sigma_{2}^{2}(W_{2}^{T}x_{2}{\left(H_{s_{1}}\right)}^{-1}x_{2}^{T}W_{2}). \end{array}$$

### A2. Derivation of bias and MSE of ${\hat{T}}_{y1}$ with sub-sampling

$$\begin{array}{cc} B_{m}\left({\hat{T}}_{y}^{*}\right)= & E_{m}(W_{s_{1}}^{T}y_{s_{1}}+W_{{\bar{s}}_{1}}^{T}x_{{\bar{s}}_{1}}b_{r}+W_{{\overset{´}{s}}_{2}}^{T}y_{{\overset{´}{s}}_{2}}+W_{{\overset{´}{\bar{s}}}_{2}}^{T}x_{{\overset{´}{\bar{s}}}_{2}}b_{2}-W_{s_{1}}^{T}y_{s_{1}}-W_{{\bar{s}}_{1}}^{T}Y_{{\bar{s}}_{1}}-W_{{\overset{´}{s}}_{2}}^{T}y_{{\overset{´}{s}}_{2}}-W_{{\overset{´}{\bar{s}}}_{2}}^{T}Y_{{\overset{´}{\bar{s}}}_{2}})\\ = & E_{m}(W_{{\bar{s}}_{1}}^{T}x_{{\bar{s}}_{1}}b_{1}+W_{{\overset{´}{\bar{s}}}_{2}}^{T}x_{{\overset{´}{\bar{s}}}_{2}}b_{2}-W_{{\bar{s}}_{1}}^{T}Y_{{\bar{s}}_{1}}-W_{{\overset{´}{\bar{s}}}_{2}}^{T}Y_{{\overset{´}{\bar{s}}}_{2}})\\ = & W_{{\bar{s}}_{1}}^{T}[x_{{\bar{s}}_{1}}E_{m}(b_{1})-E_{m}(Y_{{\bar{s}}_{1}})]+W_{{\overset{´}{\bar{s}}}_{2}}^{T}[x_{{\overset{´}{\bar{s}}}_{2}}E_{m}(b_{2})-E_{m}(Y_{{\overset{´}{\bar{s}}}_{2}})]\\ = & W_{{\bar{s}}_{1}}^{T}[x_{{\bar{s}}_{1}}\beta_{1}-x_{{\bar{s}}_{1}}\beta_{1}]+W_{{\overset{´}{\bar{s}}}_{2}}^{T}[x_{{\overset{´}{\bar{s}}}_{2}}\beta_{2}-x_{{\overset{´}{\bar{s}}}_{2}}\beta_{2}]=0 \end{array}$$

The variance of the estimator, is given by

$$\begin{array}{cc} V_{m}\left({\hat{T}}_{y}^{*}\right)= & V_{m}[W_{s_{1}}^{T}x_{s_{1}}\beta_{1}+W_{s_{1}}^{T}\epsilon_{s_{1}}+W_{{\bar{s}}_{1}}^{T}x_{{\bar{s}}_{1}}{\left(H_{s_{1}}\right)}^{-1}H_{s_{1}}\beta_{1}+W_{{\bar{s}}_{1}}^{T}x_{{\bar{s}}_{1}}{\left(H_{s_{1}}\right)}^{-1}x_{s_{1}}^{T}\epsilon_{s_{1}}\\ & +W_{{\overset{´}{s}}_{2}}^{T}x_{{\overset{´}{s}}_{2}}\beta_{2}+W_{{\overset{´}{s}}_{2}}^{T}\epsilon_{{\overset{´}{s}}_{2}}+W_{{\overset{´}{\bar{s}}}_{2}}^{T}x_{{\overset{´}{\bar{s}}}_{2}}{\left(H_{{\overset{´}{s}}_{2}}\right)}^{-1}H_{{\overset{´}{s}}_{2}}\beta_{2}+W_{{\overset{´}{\bar{s}}}_{2}}^{T}x_{{\overset{´}{\bar{s}}}_{2}}{\left(H_{{\overset{´}{s}}_{2}}\right)}^{-1}x_{{\overset{´}{s}}_{2}}^{T}\epsilon_{{\overset{´}{s}}_{2}}] \end{array}$$

$$\begin{array}{c} V_{m}\left({\hat{T}}_{y}^{*}\right)=\sigma_{1}^{2}[n_{1}+W_{{\bar{s}}_{1}}^{T}x_{{\bar{s}}_{1}}{\left(H_{s_{1}}\right)}^{-1}x_{{\bar{s}}_{1}}^{T}W_{{\bar{s}}_{1}}]+\sigma_{2}^{2}[{\overset{´}{n}}_{2}+W_{{\overset{´}{\bar{s}}}_{2}}^{T}x_{{\overset{´}{\bar{s}}}_{2}}{\left(H_{{\overset{´}{s}}_{2}}\right)}^{-1}x_{{\overset{´}{\bar{s}}}_{2}}^{T}W_{{\overset{´}{\bar{s}}}_{2}}]. \end{array}$$

Rearranging terms, we get

$$\begin{array}{c} V_{m}\left({\hat{T}}_{y}^{*}\right)=n_{1}\sigma_{1}^{2}+{\overset{´}{n}}_{2}\sigma_{2}^{2}+\sigma_{1}^{2}W_{{\bar{s}}_{1}}^{T}x_{{\bar{s}}_{1}}{\left(H_{s_{1}}\right)}^{-1}x_{{\bar{s}}_{1}}^{T}W_{{\bar{s}}_{1}}+\sigma_{2}^{2}W_{{\overset{´}{\bar{s}}}_{2}}^{T}x_{{\overset{´}{\bar{s}}}_{2}}{\left(H_{{\overset{´}{s}}_{2}}\right)}^{-1}x_{{\overset{´}{\bar{s}}}_{2}}^{T}W_{{\overset{´}{\bar{s}}}_{2}} \end{array}$$

## Supporting information

S1 Data Blood transfusion data set. (CSV)
